# Caring for children with physical disability in Kenya: potential links between caregiving and carers' physical health

**DOI:** 10.1111/j.1365-2214.2012.01398.x

**Published:** 2013-05

**Authors:** J L Geere, J Gona, F O Omondi, M K Kifalu, C R Newton, S Hartley

**Affiliations:** * Faculty of Medicine and Health Sciences, University of East AngliaNorwich; † Neuroscience Unit, Institute of Child Health, University College LondonLondon; ‡ Department of Psychiatry, University of OxfordOxford, UK; § Centre for Geographic Medicine Research (Coast), Kenya Medical Research InstituteKilifi, Kenya; ¶ Physiotherapy Department, Kilifi District HospitalKilifi, Kenya; ** Faculty of Health, University of SydneySydney, NSW, Australia

**Keywords:** Africa, caregiving, carers, developing countries, disability, health

## Abstract

**Background:**

The health of a carer is a key factor which can affect the well-being of the child with disabilities for whom they care. In low-income countries, many carers of children with disabilities contend with poverty, limited public services and lack assistive devices. In these situations caregiving may require more physical work than in high-income countries and so carry greater risk of physical injury or health problems. There is some evidence that poverty and limited access to health care and equipment may affect the physical health of those who care for children with disabilities. This study seeks to understand this relationship more clearly.

**Methods:**

A mixed methods study design was used to identify the potential physical health effects of caring for a child with moderate-severe motor impairments in Kilifi, Kenya. Qualitative data from in-depth interviews were thematically analysed and triangulated with data collected during structured physiotherapy assessment.

**Results:**

Carers commonly reported chronic spinal pain of moderate to severe intensity, which affected essential activities. However, carers differed in how they perceived their physical health to be affected by caregiving, also reporting positive benefits or denying detrimental effects. Carers focussed on support in two key areas; the provision of simple equipment and support for their children to physically access and attend school.

**Conclusions:**

Carers of children with moderate-severe motor impairments live with their own physical health challenges. While routine assessments lead to diagnosis of simple musculoskeletal pain syndromes, the overall health status and situation of carers may be more complex. As a consequence, the role of rehabilitation therapists may need to be expanded to effectively evaluate and support carers' health needs. The provision of equipment to improve their child's mobility, respite care or transport to enable school attendance is likely to be helpful to carers and children alike.

## Introduction

The health of a primary carer is a key factor which can affect the health and well-being of the child with disabilities for whom they care (Brehaut *et al*. 2004; Raina *et al*. 2005). A primary carer of a child with disabilities has been defined as the person most responsible for the day-to-day decision making and care of that child (Brehaut *et al*. 2004). Providing care or ‘caregiving’ involves assisting another person to perform activities which are necessary for survival, human functioning or social participation, or performing such activities for a person who is unable to do them (WHO 2001). Depending on the extent of assistance required and resources available, caregiving will involve variable amounts of physical work. For example, physical work may be required to assist a child with mobility, positioning or transfers, as well as for dressing, bathing, eating and drinking.

Physical work can impact variably on physical health (Walker-Bone & Cooper 2005). Physical health has been defined as that pertaining mainly to physical functioning (as opposed to emotional or cognitive functioning), physical capacity for activities or roles and bodily pain (Haley *et al*. 1994; Finch *et al*. 2002). Evidence from high-income countries has shown that the physical work and demands of caregiving can affect the physical health of carers (Raina *et al*. 2004, 2005; Murphy *et al*. 2007). For example, carers of children with physical disabilities who need assistance with transfers, have been found to have higher prevalence of back pain and decreased physical functioning compared with carers of children with chronic medical conditions who do not require such assistance (Tong *et al*. 2003, 2007).

Knowledge of the prevalence of musculoskeletal disorders such as non-specific back pain within a population is useful for service planning. However, to identify appropriate management and service provision it is also important to appreciate the impact of the type of back pain or other musculoskeletal disorders on functioning, as this may vary between individuals and specific social and cultural contexts (Waddell 2004). As pain is a multidimensional phenomenon, the impact of pain should be comprehensively evaluated in terms of pain intensity or severity, but also its relationship with functioning and the significance of pain to the individual (Anderson 2005; Dworkin *et al*. 2005). For example, Tong and colleagues (2002) found that carers' physical functioning was associated with pain severity, mood and total length of time of back pain in the previous 12 months.

In low-income countries, social and environmental factors related to the situations in which people with disabilities and their families live, influence the complex interactions between disability, poverty and health (Parnes *et al*. 2009) and this is pertinent to carers of children with disabilities in Kenya. Families of a person with disabilities tend to be poor leading to poor health (Elwan 1999). Health services are often limited and mainly available in centralized locations (Hartley 2004; Tomlinson *et al*. 2009). Therapy and equipment to assist in caregiving and mobility for people with disabilities are therefore often difficult to access, particularly for poor people and those living in remote or rural areas (Borg *et al*. 2009). Lack of access to assistive equipment may mean that caregiving in low-income countries requires more physical work and manual handling than it does in high-income countries and thus is associated with greater risk of injuries or physical health disorders. Despite a general lack of research on the physical demands of caregiving in low-income countries, there is some evidence that poverty and lack of access to health care and equipment might detrimentally affect the physical health of those who care for children with disabilities (Hamzat & Mordi 2007).

Interventions to reduce the physical work of caregiving, such as use of patient handling slings during transfers, mechanical lifts or educational programmes (Elford *et al*. 2000; Waters *et al*. 2006) can reduce injury risk in high-income countries and occupational settings. However, it should not be assumed that similar interventions will improve the physical health of carers in low-income countries (Hartley 1998, 2004) because there are many potentially important environmental, cultural, social and health-related differences between caregiving populations in these different settings.

The aim of this study was to explore the potential links between providing care of a child with moderate-severe motor impairments and the physical health of carers, in a low-income country. We also aimed to identify ways to improve the situation for carers and their families. Therefore, the study addressed the following main question (1) and sub-questions (2–5):

1. ‘How does caring for a child with moderate-severe motor impairment affect the physical health of the child's main caregiver in Kilifi, Kenya?’
2. What symptoms related to physical health do carers report?
3. Do symptoms reported by carers and observable clinical signs of impairment affecting carers indicate that they are commonly affected by particular health conditions?
4. How do carers perceive the physical demands of caregiving to be related to their own physical health?
5. How do carers think they can be supported and how can any detrimental physical effects of caregiving be reduced?

## Methods

### Study design

A mixed methods design, collecting qualitative and quantitative data, was used to identify the potential physical health effects of caring for a child with moderate-severe motor impairments. To be consistent with previous conceptualizations of physical health (Haley *et al*. 1994) and the International Classification of Functioning, Disability and Health (WHO 2001), we defined physical health of carers as that related to body structure, physical functioning (excluding emotional or cognitive functioning) and/or physical capacity for activities or role performance. This also ensured future comparability with other studies using this conceptual framework.

We recruited a purposive sample of twenty carers to achieve the following objectives:

1. Explore carers' perceptions of caregiving.
2. Collect structured interview and clinical assessment data.
3. Perform triangulation between methods of data collection and between carers.

### Study location

The study formed part of a larger project of action research looking at rehabilitation and disability in Kilifi District. The aims of the larger study were to explore the challenges faced by people with disabilities and their families and to determine sustainable ways of developing and integrating effective services into the community. The results of the larger study will be reported in separate publications.

Kilifi is a rural area on the Kenyan coast, approximately 60 km north of Mombasa. The economy in Kilifi is mainly based on subsistence farming and the district has relatively high levels of poverty (Marsh *et al*. 2010). A Kilifi Demographic and Health Surveillance System has been established in the area surrounding Kilifi District Hospital, in which information on residence, migration, births and deaths is collected through home visits conducted two to three times per year. The Kilifi Demographic and Health Surveillance System area includes approximately 240 000 people (Marsh *et al*. 2010). The study used this system to locate families of children with disabilities previously documented by members of the research team (Gona *et al*. 2006; Mung ala-Odera *et al*. 2006; Mung ala-Odera & Newton 2007).

### Sampling strategy, participant recruitment and consent

Children aged between 5 and 16 years living in the district with moderate-severe motor impairments were identified from the following sources: an existing neurological impairment database (Mung ala-Odera *et al*. 2006), a participatory rural appraisal study (Gona *et al*. 2006) and lists of health and education service users. Most children in the study were medically diagnosed as having cerebral palsy. Some had wheelchairs for seating and mobility and some were considered to be ‘bed ridden’.

We purposively selected 20 carers to include representation of a range of carer characteristics including sex, age, carer/child relationship and distance from the carer's home to therapy centres. Inclusion criteria were developed by examining the literature about the factors that have been reported to affect parental coping when they have children with physical disability (Raina *et al*. 2004; Cavallo *et al*. 2009) and/or were considered by the research team as likely to influence the experience of caregiving, its impact on physical health and therefore, the perspectives and needs of carers. The resulting inclusion and exclusion criteria used to select the sample are listed below.

Inclusion criteria were:

1. primary carers of children with moderate-severe motor impairments;
2. carers of children aged between 5 and 16 years;
3. carers with any relationship to the child; father, mother, aunt, grandmother, paid carer;
4. carers living within the study area, at variable distances from therapy departments of Kilifi District Hospital.

Exclusion criteria were:

1. people providing occasional care for a child with disabilities which was less than the amount of care provided by another individual;
2. people providing care for a child with disability (e.g. sensory or cognitive) but without moderate-severe motor impairments;
3. carers of a child with disability and under 5 years of age.

Once children had been identified, 23 primary carers (one providing care for proportionally more time than any other person) were identified and chosen through discussion between the researchers and service providers and by drawing on their knowledge of families in the region. Three carers initially recruited were subsequently excluded, as they cared for children under 5 years old. The sample's demographic characteristics are illustrated in Table 1. This sample was used for collecting both qualitative and quantitative observational data (Table 2).

**Table 1:** Demographic and structured screening questionnaire information (*n* = 20; 3 male : 17 female)

Socio-demographic information		Mean (s)	Range
Age of primary carer in years		42 (14)	24–71
Age child in years		10 (3)	5–16
Number of children living with primary carer		4 (2)	1–7
Distance to hospital from home (km)		20 (17)	0.5–50

Disability and impairment		Frequency count	Percentage (%)
Level of disability of child (moderate : severe)		7:14	33:67
Carer's disability question 1 (yes : no)*		17:3	85:15
Carer's disability question 2 (yes : no)*		0:20	0:100
Carer's musculoskeletal impairment* (yes : no)		17:3	85:15

Carer's relationship to child	*n*	Carers' general health†	*n*
Mother	13	Very good	3
Grandmother	3	Good	6
Father	2	Fair	6
Aunt	1	Poor	4
Employed caretaker	1	Very poor	1

Marital status	*n*	Type of accommodation	*n*
Married	15	Own house	11
Single	3	Rental house	4
Divorced	1	Family house	2
Separated	1	Squatter	3

* Disability and musculoskeletal impairment screening questions see data collection methods point 1.

† Carer's self-rating of own general health.

s, standard deviation.

**Table 2:** Data collection and sample size

Types of data collected	Sample
Demographic data	20
Qualitative semi-structured interview data	20
Structured physiotherapy assessment (qualitative and quantitative data)	17

The carers were contacted by a fieldworker and fully informed of the purpose and procedures of the study. A written participant information form was used and explained verbally in their preferred language. All agreed to participate. Once voluntary consent was provided demographic data were collected.

### Data collection

In this study, we used modified, existing data collection and clinical assessment tools. Three main methods for data collection were utilized (Table 2).

1. A structured questionnaire to gather information on participant demographics, disability status (of the child and the carer) and musculoskeletal impairment affecting the carer. Disability status of the children was determined by asking carers to respond with ‘Yes’ or ‘No’ to two specific questions found to be valid and reliable for detecting moderate to severe motor impairment in the region (Mung ala-Odera *et al*. 2004). A ‘Yes’ response to ‘Does your child have difficulty in holding implements, dressing and sitting upright or need help to move around?’ indicated moderate impairment and to ‘Is your child unable to walk and/or without functional use of the hands?’ indicated severe motor impairment.
  Carers responses to two questions, developed as part of ongoing work of the Washington Group on Disability Statistics (Madans *et al*. 2011) and highlighted in Finkenflugel and colleagues (2006) were used to identify whether they perceived themselves as affected by disability:
  1. Do you ever have any difficulty in doing day-to-day activities because of a physical, mental or emotional (or other) health condition which has lasted or is expected to last for 6 months or more?
  2. Do you ever need assistance in participating in any of the following activities? (walking, seeing, speaking, hearing, breathing, mental coping, learning comprehending)?
    Six questions (Atijosan *et al*. 2007) were used to identify musculoskeletal impairment affecting carers:
    1. Do you have any difficulty using your arms?
    2. Do you have any difficulty using your legs?
    3. Do you have any difficulty using any other part of your body?
    4. Do you need a mobility aid or prosthesis?
    5. Has it lasted more than 1 month?
    6. Has it, or do you expect it to last for 6 months or more?
2. In-depth interviews using an interview guide and probes which included open-ended questions about health and physically caring for their child with disabilities. Questions such as ‘What does being healthy mean to you?’, ‘Can you tell me about your experiences of caring for your child?’ and ‘How do you think caring for your child affects you?’ were used to facilitate discussion of the concepts of health in general, the relationship between caregiving and carer's health and ways that carers could be supported. The questions were piloted with a carer of two children with physical disability to ensure that translation was conceptually sound.
3. A structured physiotherapy assessment (SPA) which included structured questions (Appendix S1) and a physical assessment (Appendices S2 & S3). The SPA was developed in collaboration with Kilifi District Hospital physiotherapists and included questions about impairment, functioning, general health and medical history. The SPA also included physical assessment of range of movement, manual muscle strength testing and palpation. Assessment methods were chosen if they had been reported as having sufficient reliability for clinical or research use in peer reviewed literature and were feasible for use in the study setting (Gajdosik & Bohannon 1987; Magee 2002; May *et al*. 2006; Piva *et al*. 2006).

The structured questionnaire and semi-structured interviews were conducted by a fieldworker fluent in local language, using face-to-face interview with each carer. Questionnaire responses were manually recorded by the fieldworker or researcher and then entered into an Excel spread sheet. The interviews were fully audio-recorded, then transcribed and translated into English by two fieldworkers. A co-author (J. G.) who is a native of the area counter-checked the translations for consistency and accuracy.

Of the 20 participants, three did not report musculoskeletal impairment and were therefore not invited for physiotherapy assessment. Seventeen participants had at least one positive response to musculoskeletal impairment questions and were invited to undergo SPA (Table 2) for a more in-depth assessment of their physical health. The assessment was conducted by one of two physiotherapists, fluent in English, Swahili and local languages, in the outpatient physiotherapy department of Kilifi District Hospital. The therapists had 3 days of training, provided by the principle investigator, in assessment of musculoskeletal disorders and administration of the SPA. The SPA was piloted by each physiotherapist completing an assessment of a carer from Kilifi District, under supervision of the principle investigator. The SPA was then reviewed by the therapists and principle investigator and modified to improve the feasibility, quality and reliability of data collection.

### Data analysis

Demographic, disability and impairment data collected with the structured questionnaire were entered into SPSS (v15, IBM, USA) and descriptive statistics generated. Qualitative data were stored and managed with Nvivo software (v8, QSR International, Australia). Five transcripts were fully coded, such that all phrases or sections of transcript which were interpreted to have a particular meaning were labelled and coded as a specific ‘free node’. The coded data were then categorized into sub-themes which were further categorized into linked sub-themes until a classification of key themes was generated. This analytic process of thematically categorising the information, as described by Creswell (1998), was initially performed independently by two researchers. The independent interpretations of the data were then compared and refined until a consensus on the meaning of the data and themes generated by it was agreed. Using the agreed coding and analysis strategy the remaining 15 transcripts were fully coded and analysed by the primary investigator.

Data collected during SPA were entered into SPSS and descriptive statistics generated. The observational data gathered during SPA were used to identify the physical signs and symptoms, and therefore the type of health conditions, which might commonly affect carers. SPA data were triangulated with the themes arising from semi-structured interview data and used to answer the research questions.

## Results

### What symptoms related to physical health do carers report?

**Figure 1:**
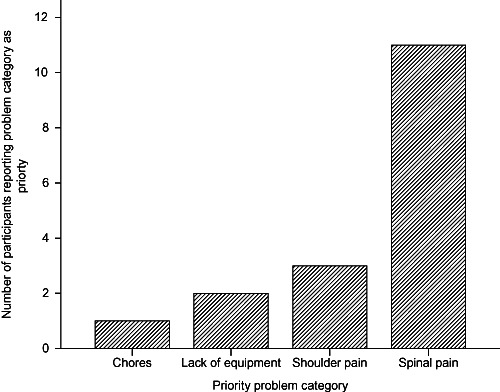
Standardized physiotherapy assessment of the carer's problem of priority.

During SPA, all carers reported chronic pain [defined as symptoms persisting for 3 months or more (Bogduk & McGuirk 2002)] as their main complaint, affecting all for more than 50% of the time in the previous month (one reporting >95% of the time). Only three reported that their symptoms were improving. Most had widespread pain in multiple areas, particularly in one or more regions of the spine and/or a limb. Spinal pain, defined in this study to include pain perceived by the participant as mainly affecting the back or thoracic regions (Bogduk & McGuirk 2002) or the cervical region (the area bordered by the lateral border of the paraspinal muscles, occipital region and seventh cervical vertebra), was identified during the SPA as a priority problem for most carers (Fig. 1). Numeric pain rating scales indicated that carers experience pain of moderate to severe intensity (Table 3).

**Table 3:** Intensity of carer's main symptomatic complaints indicated in standardized physiotherapy assessment (*n* = 17)

Variable	Mean (s)	Median (IQR)	Range
Intensity of main symptoms (at worst/10 on NPR scale)	7 (1)	7 (6–8)	5–10
Intensity of main symptoms (on average/10 on NPR scale)	5 (2)	5 (4–5)	2–7

NPR, numeric pain rating scale; s, standard deviation; IQR, interquartile range.

Carers also complained of pain during in-depth interviews, affecting a variety of body areas and including the spine. A broad variety of ‘symptoms’ were discussed, including movement dysfunction, swelling, numbness, weakness and tiredness (Table 4).

**Table 4:** Symptoms reported by carers during semi-structured interviews

Theme: carers report various symptoms related to physical health	

Sub-theme	Evidence
Pain	‘It's only my back that endures pain’ Carer 009
	‘My chest too, for instance when I carry out a tough duty then in the evening I experience pain’ Carer 016
	‘I endure pain on my hands sometimes’ … ‘On my back’ Carer 012
	‘It is my right hand’ … ‘Normally feel a throbbing pain that is so sharp and as it hurts at times it swells that I can't work anymore!’ Carer 013
Other symptoms	‘This part of my body doesn't perform well … I feel like I dislocated my arms … I feel the part losing strength’ Carer 001
	‘If I fail to perform the duties it's because of my leg the muscle sometimes swells and when it happens I fail to do the duties properly!’ Carer 016
	‘My right hand is the problem, sometimes it experience numbness’ Carer 006
	‘No it does not, the thing is, I get tired but how can I get tired yet she is my granddaughter?’ Carer 007

### Do symptoms reported by carers and observable clinical signs of impairment indicate that particular conditions commonly affect their physical health?

During SPA, all were identified as facing environmental challenges because of where they lived and were deemed by the physiotherapist to have psychosocial factors affecting their clinical presentation (Table 5). Most carers were observed during assessment to have passive and active movement restriction affecting the musculoskeletal system and all were given a provisional clinical diagnosis of musculoskeletal disorder (Table 5).

**Table 5:** Carers structured physiotherapy assessment (*n* = 17)

Variable	Frequency count(Y : N)	Percentage (%)
Carer faces environmental challenges?	17:0	100:0
Feeling generally unwell?	14:3	82:18
Weight loss?	12:5	77:23
Other illness?	4:13	20:80
Recent operations?	1:16	6:94
Taking medication?	12:5	71:29
Family history of illness?	8:9	47:53
Cauda equina indicator absent	17:0	100:0
Psychosocial factors present	17:0	100:0
Active movement restriction	12:5	71:29
Passive movement restriction	13:4	77:23
Structured physiotherapy assessment diagnosis of musculoskeletal disorder	17:0	100:0

During SPA, most carers also reported feeling generally unwell, recent weight loss, and that they were taking medication (Table 5). These aspects of the patient reported history are commonly used as indicators for the potential presence of systemic or serious pathology other than simple musculoskeletal disorders (Greene 2001). The potential importance of such symptoms appears not to have been reflected in the clinical diagnosis resulting from the SPA, despite training in use of the SPA to identify the potential presence of more serious conditions.

### How do carers perceive the physical demands of caregiving to be related to their own physical health?

**Figure 2:**
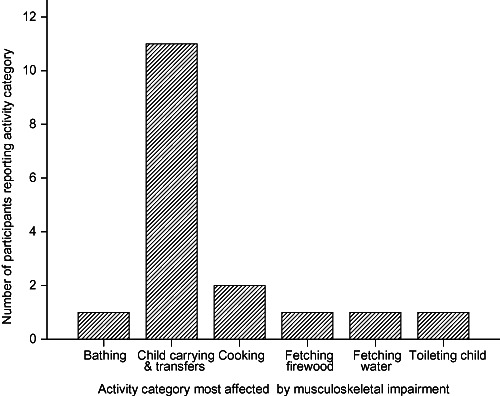
Activities most affected by impairment.

Carers described the physical difficulties they faced in caring for their child, emphasising difficulties carrying their child both in semi-structured interviews (Table 6) and SPA (Fig. 2).

**Table 6:** Relationships between demands of caring and physical health

Theme: physical difficulties	

Sub-theme	Evidence
Difficulty carrying as child grows	‘I use to take her but I don't take her anymore because she is overweight and every time I have to carry her and sometime the vehicle are full and so I do the therapy for her’ Carer 003
	‘she is now old and with weight so you can't lift her for long’ Carer 006
	‘the problem is whenever I travel I have to carry her’ Carer 022
Physical symptoms linked to caring tasks	‘Also he's not the kind of child, who is stiff enough, yet he uses force and so you have to hold him so tightly because if you don't he falls. That's when I used to experience pain on my ribs. It's better now because I don't do it often and the ribs stopped because he used to get hold of my ribs tightly’ Carer 002
	‘for the exercise you have to go to the hospital that's when I endure pain on my shoulder because I carry her on my back’ Carer 003
	‘I get tired’ Carer 007
	‘because I'm with him from morning to evening then I only get tired’ Carer 005
Physical symptoms linked to difficult tasks	‘My chest too, for instance when I carry out a tough duty then in the evening I experience pain’ Carer 016
	‘Maybe if I have done hard task when I feel pain but with the normal duties I do not have any problem’ … ‘I undergo pain on my waist and my hand gets tedious’ Carer 022
No physical difficulties	‘I have never felt anything in my body’ Carer 014
	‘to be frank I don't think I have any problem I'm physically fit’ Carer 005

During SPA carers were asked to describe the activity most affected by the main symptoms which they had reported and most indicated child carrying and transfers as most affected (Fig. 2). Carers were asked to rate the level of importance of the activity which was most affected by their symptoms, choosing from (1) not important at all; (2) slightly important; (3) very important; or (4) essential activity. All carers rated the activity most affected by musculoskeletal impairment as being an essential activity, other than one carer who rated child carrying and transfers as very important.

When physical health was discussed in semi-structured interviews, pain in various body regions was at times described in relation to tasks performed to care for their child suggesting that carers perceived an association between caring and symptoms (Table 6). However, carers also discussed symptoms related to other unspecified or ‘tough’ tasks (Table 6). Some carers denied awareness of any effect on their own physical health from caregiving or reported that they were physically well.

However, other effects of caregiving not directly related to physical health were raised in semi-structured interviews (Table 7). Both positive effects, such as feeling ‘good in my heart’ and negative effects, such as guilt, were discussed. Carers also talked about caring as a blessing through God's work and described difficulties in performing activities and work (Table 7).

**Table 7:** Relationships between demands of caring and other aspects of health and well-being

Theme: non-physical domains of health are affected by caring	

Sub-theme	Evidence
Positive effects on emotions	‘It improves my life for example when I wash and feed him he is happy he laughs and so I feel good in my heart’ Carer 005
Negative effects on emotions and mood	‘It affects because you will not be happy you feel sad in your heart and so your health is affected’ Carer 016
	‘She is the child I gave birth to but still it hurt in my heart to see her in that condition’ Carer 009
	‘My health yes, I feel pity in my heart because she cannot walk’ Carer 015
	‘The child cannot carry out anything for herself she cannot give support to herself so I sometime sit down and reflect a lot. Also I wonder where I/ the child went wrong so I feel guilty. Sometimes a person might come and ask “hasn't the child been able to walk since that time? What are you going to do?” then I say nothing because there is nothing to be done, but that makes me feel terrible in my heart and I can even put aside whatever I was doing just to go to bed because I feel horrible in heart!’ Carer 023
Caring is a blessing through God's work	‘It becomes good in that by taking care of her God blesses us’ Carer 014
	‘My life keeps on going well because we were given that child by God and we accept him because he's like the kid who is walking and you have accepted just like you are told to accept Aids so if you accept such a thing then your life becomes good’. Carer 002
Difficulty performing activities and work	‘The effect is; I cannot be away to do my own duties like working because I cannot leave her alone’ Carer 022
	‘There are a lot of activities outside that I cannot do if I'm alone with the child I cannot fetch water/ go to the market since you have fail to go to the shamba (farm) will you go to the market? So you remain here’ Carer 008

### How might carers be supported?

In the semi-structured interviews, carers suggested ways in which their needs could be supported and their situation improved (Table 8). They focussed on support in two key areas which might reduce their physical burden of care. Firstly, enabling children to attend school, or when they remain at home, providing some additional supervision. This would free up time for other activities and potentially reduce the physical work of caring for the child during their school attendance time. Removing barriers to school attendance may therefore not only benefit the child with disabilities, but also their primary carer and as a consequence other siblings.

**Table 8:** How to support carers

Theme: supporting carers	

Sub-theme	Evidence
School attendance and child minding	‘I don't know but if there could be a support for my child to be taken to school I can even get relief. I took her once but she did not get any support so I brought her back that's why I cannot say what kind of help I need but all I need the most is for my child to attend school!’ Carer 016
	‘I need at least if there was anyone to take a look at the child so as to do my duties’ Carer 012
	‘I wish she gets helps as to be able to go to school’ Carer 022
Food and income	‘OK during this time when I'm taking care of my baby and when things are not ready and when food is not enough for the family that's when my health is affected. But during good times I don't experience many problems’ Carer 001
Equipment	‘Just the wheelchair because she will be able to go to school since it is tiresome for me to be carrying her everyday’ Carer 009
	‘My child is unable to walk and so if she get the callipers then she can even go to the toilet on her own, she is able to hold a cup but the problem is that the legs lack strength. Also if she can get the wheel chair, then I can even go with her anywhere I want because if she is alone at home then I cannot be paying special attention in whatever I will be doing/wherever I will be’ Carer 023
Access to therapy	‘by God willing if she also gets the full therapy to make her legs strong to be able to walk then I will appreciate a lot!’ Carer 023
	‘There are things like therapy because for now she is not attending any therapy so I wake up very early to do the therapy for her and then I put her on bed since there is no one to assist and give her support to sit on the chair, therefore it's like I do nothing’ Carer 006

Secondly, carers highlighted the need for suitable equipment, such as wheelchairs or callipers, but also simply shoes, to facilitate their child's mobility and reduce the need for carrying. Pain was particularly attributed to carrying heavier children and the inability to carry children over distance was discussed as a barrier to attending therapy or school.

One mother raised the issue of support through additional income or food. While some indicated that access to medication or therapy and an improvement in their child's physical impairment would help, the importance of understanding between the child's parents was also highlighted by one carer as a means to coping with any difficulties.

## Discussion

Despite raising other health-related issues, such as tiredness or the emotional aspects of caregiving, carers in this study most commonly complained of pain in a variety of areas and in the SPA focussed on spinal pain. Health issues highlighted by participants were similar to those which were found by Tong and colleagues (2002) to be significantly associated with physical functioning; pain was usually chronic and of moderate to high intensity, with some participants discussing low mood in the context of their child's situation and caregiving. Our findings indicate that the impact of the symptoms experienced by participants in this study was high, mainly affecting essential caregiving activities.

The physiotherapists in this study most often interpreted carers' complaints of pain, as being suggestive of simple musculoskeletal disorders, such as non-specific low back pain or shoulder pain. However, bodily pain combined with reported weight loss, feeling unwell and medication consumption, as described in Table 5, may also be indicative of underlying serious or systemic illness such as fracture, infection or cancer (Deyo *et al*. 1992; Siemionow & McLain 2006). Similarly, chronic joint, muscle or neuropathic pain can be a feature of HIV (Gray & Berger 2007) and may signal the presence of this or another co-morbidity.

This suggests that the health needs of carers such as those in our study may be complex, co-morbidities may be difficult to identify or poorly recognized and that health care professionals dealing with carers may need considerable skill in the differential diagnosis of musculoskeletal pain. Existing co-morbidity may not be apparent to a physiotherapist or health worker if information is collected and interpreted through routine clinical assessment. A more detailed and holistic understanding of the carer's health status may be required for appropriate advice and safe treatment to be offered.

This highlights a potential area of need for staff capacity building in Kilifi. For example, additional training for therapy staff in the assessment of chronic pain and appraisal of carers' needs could extend their scope of practice by enabling them to identify when referral for further investigation or other treatment is required. More comprehensive assessment may lead to more accurate diagnoses and thorough needs assessments, and enable therapists to identify a broader range of appropriate therapies which they or other health professionals could provide.

Some carers linked the physical dysfunction or symptoms which they experienced to caregiving activities. However, this does not demonstrate a causal link, as robust epidemiological evidence is required to do this (Beaglehole *et al*. 1993). Yet our study does indicate that carers commonly experience physical health or mobility problems while carrying out routine yet essential physical tasks required for care of their child with disabilities. Carers did not offer a wide variety of suggestions about how they might be better supported, but did focus mainly on improvements to their child's mobility. Carers also focussed on enabling their child to attend school to create respite or opportunities for them to engage with other work. These results imply that timely provision of appropriate equipment or respite from caregiving activities would reduce the burden of physical care and positively influence carers' health. Again, therapists could expand their role, for example, through lobbying for better provision of equipment, transport or respite care to facilitate access to schools and health services.

How best to provide appropriate equipment to improve children's mobility or facilitate school attendance then becomes an important question. However, particularly where funding for service improvements is limited, supporting and training families and community members to produce inexpensive equipment from locally available materials could enable them to more independently meet their needs. Community-based rehabilitation has been suggested in the literature as a viable mechanism through which to achieve this (Finkenflugel *et al*. 2006; Hartley *et al*. 2009) and has the potential to link health services to community activities and more effectively tailor them to carers needs (Hartley 1998, 2004, 2006). Therapists may also play an important role in training community-based rehabilitation workers to provide therapy, to reduce the burden of carrying children with disabilities while travelling to distant centres.

However, a clear link between caring for a child with disabilities and carers own health-related problems or symptoms, was not made in all cases. Some carers denied any negative impact (Table 6) or described positive impacts of caring on their own health, such as feeling good, or being ‘blessed by God’ (Table 7). Our study illustrates that carers' perceptions of their own health are formed as part of a complex situation and influenced by opportunities for respite and work as well as emotions and spiritual beliefs. Along with accurate clinical diagnosis to exclude serious disease, a holistic approach to assessment of carers' needs seems to be required as physiotherapists also felt that all participants were affected by psychosocial issues and environmental challenges (Table 5). Others have found a holistic bio-psychosocial approach to be important in chronic pain management (Waddell 2004) and for supporting carers in higher-income settings (Raina *et al*. 2004). This may also require capacity building and training for rehabilitation therapists, for example, in the use of effective communication and ‘talking’ therapies or better recognition and use of existing skills within therapy teams. It also indicates that therapists must have better comprehension of disability as an interaction between person with disabilities, their family, community and environment.

The literature has previously shown that carers can use existing family and social networks for respite and support (Hartley *et al*. 2005). As one participant in our study stated;

If both parents understand each other, then nothing will affect you. There are others when I used to go to therapy really, I felt pity for them, for instance such a child is left with her grand mum while the parents decide to separate and that breaks your heart. If both parents accept the situation then the child lives just like any other normal child (Carer 002)

Improved communication and understanding of disability issues among family members, women's groups and community groups, may encourage people to provide respite through physical assistance or social and emotional support for carers. The narrow focus of carers' suggestions for support implies that educational interventions for carers may also be useful. Workshops or opportunities for carers of children with disabilities to meet could enable them to share experiences and skills and develop creative ideas for developing additional local support strategies (Ellis 2010).

### Limitations of the study

This study has increased understanding of the complexity of health issues affecting carers of children with disabilities in Kenya, but was not able to determine whether the symptoms reported by participants were ‘caused’ by the additional work of caregiving, mainly because the sample was small and purposive. This was appropriate for the predominantly qualitative approach and the limited time frame and resources available. It was also not determined whether the data analysis had reached a point of saturation, as the time and resource constraints of the study did not allow for any further data collection and analysis. A limitation of the purposive sampling strategy is that it does not allow for inferential statistical analysis and generalization to a larger population. A future study might consider using a randomized sampling process and compare them with a second group of carers of children without disabilities, to see if they too had pain and back problems.

A pragmatic approach was taken to utilize existing physiotherapy services and resources to perform clinical assessments. Comprehensive medical and radiological investigations could have provided more detailed diagnostic information, but would have involved greater cost and burden on existing services as well as study participants. Our aim was to use the findings of clinical assessments as they can be performed by providers of therapy services in Kilifi, together with data from semi-structured interviews. These methods of data collection provided insight into carers' health without the need for additional diagnostic tests, which would normally be inaccessible to therapy providers. To establish the statistical level of reliability of the clinical assessment protocol used in this study, each participant would need to undergo full clinical assessment twice. This would impose a substantial burden on the study participants and resources, so methods shown to have sufficient reliability for clinical and research use in other settings were used in the protocol. We provided training and piloted the assessment methods to improve quality and reliability of data collected.

## Conclusions

Carers of children with moderate-severe motor impairment in Kilifi report problems with their own physical health which may impact on their capacity for physical functioning and to care for their child. Carers in this study reported symptoms typical of musculoskeletal disorders, particularly spinal pain. Support with the physical work of caregiving may, therefore, be a way to improve the physical health of carers and the health and well-being of children with disabilities. However, reports of spinal pain may also indicate the presence of other medical conditions and expanded roles for rehabilitation therapists may be useful to optimize service provision in supporting carer health and physical functioning.

**Key messages**

- Carers of children with moderate or severe motor impairment in Kilifi report problems with their own physical health which impact on their capacity for physical functioning and to care for their child.
- Carers commonly complained of moderate to severe, chronic spinal pain.
- While routine assessments lead to diagnosis of simple musculoskeletal pain syndromes, the overall health status and situation of carers may be more complex.
- Capacity building may enable therapists and health care workers to more effectively meet carers' needs.
- The provision of simple equipment to improve their child's mobility and respite care or transport to enable children with disabilities to attend school is likely to be helpful to carers and children alike.

## Supporting information

**Appendix S1.** Standardized physiotherapy assessment.

**Appendix S2.** Spinal physical assessment.

**Appendix S3.** Peripheral joint physical assessment.
