# Supplementary material for: Caring for children with physical disability in Kenya: potential links between caregiving and carers' physical health
Source: Child Care Health Dev. 2013 May;39(3):381–92. doi: 10.1111/j.1365-2214.2012.01398.x (PMC3654176; doi:10.1111/j.1365-2214.2012.01398.x)
Supplement: Supplementary file 1 [file cch0039-0381-SD1.doc]

**Appendix 1: Standardised Physiotherapy Assessment (SPA)**

**Subjective Assessment: Kilifi Hospital 2008**

| **Name** |  |
| --- | --- |
| **Participant Number** |  |
| **Age or date of birth** |  |
| **Contact details** |  |

**1 Environment:**

**1a** ‘Can you tell me what it is like where you live?’ (e.g. rural or urban, type of housing, who do you live with, access to facillities such as schools shops, etc)

**1b** ‘Do you face any challenges because of where you live?’ Yes 🞏 No 🞏

(if so, what are they?)

**1c** ‘What are the good things about where you live?’

**2 Activities and Participation:**

**2a** ‘What activities do you normally perform or participate in?’ (please list)

**2b ‘What are you unable to do or do you have difficulty doing (because of your physical problem(s))?’**

(describe in boxes below next to Activity 1:, 2:, 3: etc)

**2c ‘How important is each activity to you?’**

(participant to circle level of importance to them 0 – 10)

Activity 1:

1. not important at all 🞎 2. slightly important 🞎

3. very important 🞎 4. essential activity 🞎

Activity 2:

1. not important at all 🞎 2. slightly important 🞎

3. very important 🞎 4. essential activity 🞎

Activity 3:

1. not important at all 🞎 2. slightly important 🞎

3. very important 🞎 4. essential activity 🞎

Activity 4:

1. not important at all 🞎 2. slightly important 🞎

3. very important 🞎 4. essential activity 🞎

Activity 5:

1. not important at all 🞎 2. slightly important 🞎

3. very important 🞎 4. essential activity 🞎

**2d List the activites you have difficulty doing or are unable to do (2b), in order of what is most important to you:** (From most important activity to least important activity)

**Area of symptoms (describe all reported symptoms):**

**Any pins and needles, numbness or altered sensation?** Yes 🞏 No 🞏

**3a Area of symptoms (describe all reported symptoms):**


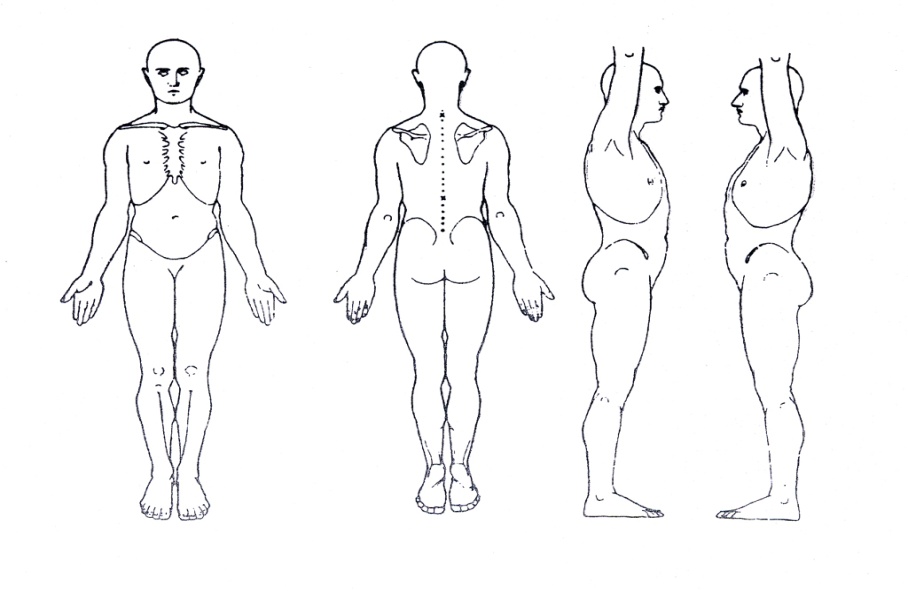
 **3b Any pins and needles, numbness or altered sensation?** Yes 🞏 No 🞏

Anterior

Posterior

Right

Left

**(If so please indicate which and location on body chart)**

**3c Cauda Equina indicators**

Do you have any problems with your bladder or bowel? Yes 🞏 No 🞏

**3d Most troublesome (main) symptom =**

**3e Severity of main symptom when at its worst (please circle):**

*0 = No symptom 10 = symptom as bad as you can imagine*

0 1 2 3 4 5 6 7 8 9 10

**3f Severity of main symptom on average over past week (please circle):**

0 1 2 3 4 5 6 7 8 9 10

**3g In the past 30 days, main symptoms occurred:**

<25% of time <50% time =/>50 % time >95% time

**3h 24 Hour behaviour of main symptom:**

AM: Better 🞏 Unchanged 🞏 Worse 🞏

DAY: Better 🞏 Unchanged 🞏 Worse 🞏

NIGHT: Better 🞏 Unchanged 🞏 Worse 🞏

**4 Impairment**

**4a** ‘What is it that *most* affects the activity that is *most* important to you?’ (Only if more than one symptom reported)

***If** the symptom which most affects the most important acitivity (e.g. arm pain affecting stirring food), is different than the most troublesome (main) symptom (3d e.g. back pain) determine which is the greater problem for the patient:

**4b ‘What is the biggest issue for you; the most troublesome (main) symptom or the most restricted activity?’**

The following questions relate to either the most important restricted activity, or to an activity which affects the most troublesome symptom. If they are different but equally important, complete questions **4c-8** twice.

**4c Aggravating factors**

‘Can you do (most important activity) at all?’ Yes 🞏 No 🞏

If so: ‘How long for?’

‘What is it that makes you stop?’

‘Once you stop the activity how long does it take to settle?’

**4d Easing factors**

‘Is there anything you can do to make this activity easier or reduce your symptoms?’ Yes 🞏 No 🞏

If yes: ‘What do you do?’

**5 Past History**

**5a** ‘Have you had this problem in the past, before this episode started?’

Yes 🞏 No 🞏

**5b** If so: ask for detail of how/why it started, when, how often it has occurred

**6 Current History: Most Recent Episode**

**6a** ‘When did you start to have this problem, (this time)?’__________________

**6b** ‘Has it changed? (if no skip 6c)* Yes 🞏 No 🞏

**6c** Is it better or worse?’* **Better** 🞏 **Worse** 🞏

* additional explanation if required:

**6d** ‘How did the problem start (this time)?’

**6e** ‘What do you believe has caused this problem (this time)?’

**7 Previous treatment**

**7a** ‘Have you tried any treatment so far? Yes 🞏 No 🞏

**7b** ‘Did it help?’ Yes 🞏 No 🞏

**7c** ‘What was the treatment?’

**8a** ‘Have you had any medical investigation for this problem?’ Yes 🞏 No 🞏

**8b** Investigations:

**9 General health**

**9a** ‘Are you feeling well in yourself generally?’ Yes 🞏 No 🞏

**9b** ‘Have you lost weight recently?’ Yes 🞏 No 🞏

**9c** ‘Do you have any other illness or medical conditions?’ Yes 🞏 No 🞏

Other illness:

**9d** ‘Have you had any recent operations?’ Yes 🞏 No 🞏

Operation for:

**9e** ‘Do you have a history of certain illness in your family?’ Yes 🞏 No 🞏

Family illnesses:

**9f** ‘Are you taking any medicine?’ Yes 🞏 No 🞏

Medication:
