# Supplementary material for: Caring for children with physical disability in Kenya: potential links between caregiving and carers' physical health
Source: Child Care Health Dev. 2013 May;39(3):381–92. doi: 10.1111/j.1365-2214.2012.01398.x (PMC3654176; doi:10.1111/j.1365-2214.2012.01398.x)
Supplement: Supplementary file 2 [file cch0039-0381-SD2.doc]

**Appendix 2: Spinal Region^1^: Date: Participant name:**

| **Observation^2^:**  **Patient demonstration of Sx prod:** | | | | | | |
| --- | --- | --- | --- | --- | --- | --- |
| **Active Movement**  Direction | **Range**^3^  Degrees°/ 🡫’d  FROM= 🗸  FROM+OP= 🗸🗸 | | **Sx reproduction?** ^4^  Produced (Pr), 🡩 or 🡫  (which Sx from body chart) | | **Sx**^5^ **alterable?**  Yes/No  (how) | |
| **Flexion** |  | |  | |  | |
| **Extension** |  | |  | |  | |
| **Rotation L** |  | |  | |  | |
| **Rotation R** |  | |  | |  | |
| **Passive Accessory Movement** | | **Sx reproduction^4^?**  Yes/No: If yes state which symptom | | | | **Mobility^6^**  🡫 or 🡩 |
| **PA central** | |  | | | |  |
| **Palpation^7^** | | | | **Neurological tests** | | |
| **Other findings** (describe)**:**  **Location:** | | | | **Sensation**^8^**:**  **Power**^9^**:**  **Reflexes**^10^**:**  **SLR Sx prod**^11^**: Yes 🞎 No 🞎 N/A 🞎** | | |
| **Special tests^12^:**  **Tx Symptom with full inspiration: Yes 🞎 No 🞎 N/A 🞎**  **SIJ Symptom reproduction with: Distraction Yes 🞎 No 🞎 N/A 🞎**  **Thigh Shear Yes 🞎 No 🞎 N/A 🞎**  **Compression Yes 🞎 No 🞎 N/A 🞎**  **PA pressure Yes 🞎 No 🞎 N/A 🞎** | | | | | | |
| **Provisional diagnosis^13^:** | | | | | | |

**Areas screened:**

**Problem List:**

| **Problem** | **Priority of problems**  **(1= highest)** |
| --- | --- |
|  |  |
|  |  |
|  |  |
|  |  |
|  |  |

**Intervention (e.g. advice given or exercises demonstrated):**

**Key to Spinal Physical Assessment Form**

**^1^ Spinal region:** Cervical, lumbo-pelvic or thoracic

**^2^Observation:** describe any obvious deformity such as scoliosis, kyphosis, asymmetry of posture or muscle bulk

**^3^ Range of motion**

| **Region** | **Active movements to assess** |
| --- | --- |
| **Lumbar** | Flexion in standing |
|  | Extension in standing |
|  | Rotation left in sitting |
|  | Rotation right |
| **Thoracic** | Flexion in sitting |
|  | Extension in sitting |
|  | Rotation left in sitting |
|  | Rotation right in sitting |
| **Cervical** | **Flexion (measure in degrees°) in sitting** |
|  | **Extension (measure in degrees°) in sitting** |
|  | **Rotation left (measure in degrees°) in supine lying** |
|  | **Rotation right (measure in degrees°) in supine lying** |

- for **cervical spine** indicate range measured in degrees with gravity goniometer or indicate whether full range of motion (🗸) or full range of motion with over pressure (🗸🗸)
- for **lumbar spine and thoracic spine** indicate if decreased range (🡫) (these regions not measured in degrees) or indicate whether full range of motion (🗸) or full range of motion with over pressure (🗸🗸)

**^4^Are any symptoms reproduced, increased or decreased with active or passive movement?** If so, indicate which one by using the same label as on the body chart, indicate whether produced (**pr**) increased (🡩) or decreased (🡫).

**^5^If symptom is reproducible with active movement, does it change** e.g. with:

- Correction of postural deviation/changing posture
- Change to muscle recruitment pattern
- Manual therapy technique such as a passive accessory glide during movement
- Repeated movements

**^5^ Mobility:** this is a qualitative judgement, does there appear to be more or less movement during accessory mobilisation compared to other levels of the spine in that region, or between the right and left side, or to what is considered ‘usual’ for someone of the same age?

| **LEFT SIDE OF BODY** | **RIGHT SIDE OF BODY** |
| --- | --- |

**^7^ Palpation:** is anything else (other than pain) detected on palpation, for example warmth, lumps or bumps, swelling, muscle spasm

**^8^Neurological testing of sensation:** light touch and pin prick: touch and point technique. Is there any reduction of sensation, or a difference in sides?

**^9^ Neurological testing of power:** myotomal isometric muscle contraction to evaluate spinal nerve conduction deficit

| **Cervical spine and upper limb** | | |
| --- | --- | --- |
| **Myotomal level** | **Muscle action tested isometrically (no movement occurs)** | **Reflex** |
| C1 | Upper cervical flexion |  |
| C2 | Upper cervical extension |  |
| C3 | Cervical lateral flexion |  |
| C4 | Shoulder girdle elevation |  |
| C5 | Shoulder abduction | Biceps jerk |
| C6 | Elbow flexion | Biceps jerk |
| C7 | Elbow extension | Triceps jerk |
| C8 | Thumb IP joint extension; finger DIP flexion |  |
| T1 | Finger abduction and adduction |  |
| **Lumbar spine and lower limb** | | |
| L2 | Hip flexion |  |
| L3 | Knee extension | Knee jerk |
| L4 | Foot dorsiflexion and inversion | Knee jerk |
| L5 | Extension of big toe |  |
| S1 | Foot plantarflexion standing (or eversion of the foot) | Ankle jerk |
| S2 | Toe standing (or knee flexion) | Ankle jerk |

**^10^** Includes testing of **deep tendon reflexes (in table above), clonus and babinski**

**^11^ SLR: ‘**straight leg raise’ to evaluate mechanosensitivity of the sciatic nerve; the relaxed limb is passively raised by the assessor, producing hip flexion while maintaining knee extension to the onset of symptoms.

**^12^** **Special tests for particular symptoms in thoracic or pelvic region**.

| **Region** | **Test** |
| --- | --- |
| Sacroiliac joint | Any 2 of 4 tests reproduce pain: distraction, femoral shear, compression, postero-anterior pressure on sacrum= SIJ problem; 0/1 painful= not SIJ problem |
| Thoracic | Reproduction of symptoms with deep inspiration (deep breath) |

**^13^** **Provisional diagnosis** given the information currently available to assessor categorise participant’s presentation as either:

- Non-specific musculoskeletal disorder
  - Identified by region of disorder: back, neck, thoracic or pelvic
  - And identified by nature of dysfunction e.g. pain or hypomobility (stiffness)
- Potentially serious condition
  - Identify indicators and classify as potentially systemic, infectious disease or unstable neurological condition
