# Supplementary material for: Caring for children with physical disability in Kenya: potential links between caregiving and carers' physical health
Source: Child Care Health Dev. 2013 May;39(3):381–92. doi: 10.1111/j.1365-2214.2012.01398.x (PMC3654176; doi:10.1111/j.1365-2214.2012.01398.x)
Supplement: Supplementary file 3 [file cch0039-0381-SD3.doc]

**Appendix 3: Body Region^1^: Date: Participant name:**

| **Observation^2^:**  **Patient demonstration of Sx prod:** | | | | | | | | | | | | | | | |
| --- | --- | --- | --- | --- | --- | --- | --- | --- | --- | --- | --- | --- | --- | --- | --- |
| **Active^3^ Movement**  Direction | | **Range^4^**  Deg°, cm or 🡫  FROM= 🗸  FROM+OP= 🗸🗸 | | | **Sx prod?^5^**  Yes/No  (which) | | | | **Strength^6^**  **MRC**  grade 0-5  or PI | | | | **Control^7^**  Poor (P)/Good (G) | | |
|  | |  | |  |  | | |  |  | |  | |  | |  |
|  | |  | |  |  | | |  |  | |  | |  | |  |
|  | |  | |  |  | | |  |  | |  | |  | |  |
|  | |  | |  |  | | |  |  | |  | |  | |  |
| **Isometric muscle^8^ contraction**  Reproduces symptoms? (Y/N) | | | **Passive^9^ Movement**  Direction | | | | **Passive Range^10^**  = or > AROM  FROM: 🗸 | | | | | **Sx production?^5^**  Yes/No  (which) | | | |
|  |  | |  | | | |  | | |  | |  | |  | |
|  |  | |  | | | |  | | |  | |  | |  | |
|  |  | |  | | | |  | | |  | |  | |  | |
|  |  | |  | | | |  | | |  | |  | |  | |
| **Capsular pattern^11^? Yes No** | | | | | | | | | | | | | | | |
| **Palpation^12^** | | | | | | **Neurological tests** | | | | | | | | | |
| **Symptom reproduction? Yes No**  **Location:**  **Other** (describe)**:**  **Location:** | | | | | | **Sensation^13^:**  **Power^14^:**  **Reflexes^15^:**  **Provocation tests^16^:** | | | | | | | | | |
| **Special tests^17^:** | | | | | | | | | | | | | | | |
| **Provisional Diagnosis^18^:** | | | | | | | | | | | | | | | |

**Areas screened:**

**Problem List:**

| **Problem** | **Priority of problems**  **(1= highest)** |
| --- | --- |
|  |  |
|  |  |
|  |  |
|  |  |
|  |  |

**Intervention (e.g. advice given or exercises demonstrated):**

**Key to Physical Assessment Form**

**^1^Body region:** Indicate which of hip, thigh, knee, leg, ankle and foot, shoulder, arm, elbow, forearm or wrist and hand

**^2^Observation:** describe any obvious deformity such as spinal scoliosis, kyphosis, missing limb or digit, joint contracture, asymmetry of posture or muscle bulk

**^3^Active & ^9^Passive Movements to assess:**

**^4^AROM**: record in degrees or centimetres measured with goniometer or as decreased (🡫)

**^10^PROM: record as equal to (=) or greater than (>) AROM or full range of motion (✓)**

|  | **Active Range of Motion (AROM)** | **Passive Range of Motion (PROM)** |
| --- | --- | --- |
| **Shoulder** | **Abduction (n=180°)** | Abduction/quadrant (>AROM;= AROM; ✓) |
|  | **Flexion (n=180°)** | Flexion (>AROM;= AROM; ✓) |
|  | **External rotation (n=90° in 90°abd)** | **External rotation in supine 20° abd (°)** |
| **Elbow/**  **forearm** | **Flexion (n=150°)** | Flexion (>AROM;= AROM; ✓) |
|  | **Extension (n=0°)** | Extension (>AROM;= AROM; ✓) |
|  | Pronation in 90° EF (n=80-90°) | Pronation in 90° EF (>AROM;= AROM; ✓) |
|  | Supination in90° EF (n=80-90°) | Supination in 90° EF (>AROM;= AROM; ✓) |
| **Wrist** | **Flexion (n=80°)** | Flexion (>AROM;= AROM; ✓) |
|  | **Extension (n=70°)** | Extension (>AROM;= AROM; ✓) |
|  | Radial deviation (decreased/FROM 20°) | Radial deviation (>AROM;= AROM; ✓) |
|  | Ulnar deviation (decreased/FROM 30°) | Ulnar deviation (>AROM;= AROM; ✓) |
| **Hand** | Power Grip/Fist (decreased or FROM) | MCP, PIP, DIP flexion (>AROM;= AROM; ✓) |
|  | **Thumb/finger tip opposition**  **(n=0cm)** | MCP, PIP, DIP extension  (>AROM;= AROM; ✓) |
| **Thumb** | **CMC Extension (n=20°)** | Extension (>AROM;= AROM; ✓) |
|  | **CMC Abduction (n=70°)** | Abduction (>AROM;= AROM; ✓) |
| **Hip** | **Flexion (n=120°)** | Flexion (>AROM;= AROM; ✓) |
|  | **Abduction (in neutral F/Ext) (n=45°)** | Abduction (neutral F/Ext)  (>AROM;= AROM; ✓) |
|  | **Internal rotation (in 90° F) (n=45°)** | Internal rotation (in 90° F)  (>AROM;= AROM; ✓) |
|  | **External rotation (in 90° F) (n=45°)** | External rotation (in 90° F)  (>AROM;= AROM; ✓) |
| **Knee** | **Flexion (n=135°)** | Flexion > or = AROM (>AROM;= AROM; ✓) |
|  | **Extension (n=0°)** | Extension > or = AROM  (>AROM;= AROM; ✓) |
| **Ankle** | **Dorsiflexion (standing knee-wall cm)** | Dorsiflexion > or = AROM  (>AROM;= AROM; ✓) |
|  | **Plantarflexion (n=50°)** | Plantarflexion > or = AROM  (>AROM;= AROM; ✓) |

| **LEFT SIDE OF BODY** | **RIGHT SIDE OF BODY** |
| --- | --- |

**^5^Are any symptoms reproduced, increased or decreased with active or passive movement?** If so, indicate which one by using the same label as on the body chart, indicate whether produced (**pr**) increased (🡩) or decreased (🡫).

**^6^MRC scale (modified*) for testing muscle strength**

| **Grade** | **Muscle activity** |
| --- | --- |
| 0 | No contraction |
| 1 | Flicker or trace of contraction |
| 2 | Active movement with gravity eliminated |
| 3 | Active movement against gravity |
| 4 | Active movement against gravity and resistance |
| 5 | Normal strength |
| PI* | Muscle contraction/movement inhibited by pain |

**^7^This is a qualitative judgement**: Does the person have any indicators of **poor** movement co-ordination or control (shaking, tremor, trick movements) or indicators of **good** movement control (smooth, even paced movement)?

**^8^Isometric muscle contraction:** does it reproduce symptoms (e.g.pain)

**^11^Capsular patterns of motion restriction**

| **Joint** | **Movement restriction** |
| --- | --- |
| Shoulder joint | Lateral rotation restriction greater than (>) abduction restriction>medial rotation restriction |
| Elbow | Flexion restriction>extension restriction |
| Wrist | Flexion=extension |
| CMC thumb | Full flexion; limited abduction and extension |
| Thumb & fingers | Flexion>extension |
| Hip | Medial rotation restriction> extension restriction> abduction restriction> flexion restriction> lateral rotation restriction |
| Knee | Marked flexion limitation, slight extension limitation |
| Ankle | Plantarflexion restriction> dorsiflexion restriction |
| MTP great toe | Extension restriction> flexion |
| MTP toes 2-5 | Tends to fix in extension with IP joint flexion |

**^12^Palpation:** is anything else (other than pain) detected on palpation, for example warmth, lumps or bumps, swelling, muscle spasm

**^13^Neurological testing of sensation:** light touch and pin prick

touch and point technique

**^14^Neurological testing of power:** myotomal isometric muscle contraction to evaluate spinal nerve conduction deficit

| **Cervical spine and upper limb** | | |
| --- | --- | --- |
| **Myotomal level** | **Muscle action tested isometrically (no movement occurs)** | **Reflex** |
| C1 | Upper cervical flexion |  |
| C2 | Upper cervical extension |  |
| C3 | Cervical lateral flexion |  |
| C4 | Shoulder girdle elevation |  |
| C5 | Shoulder abduction | Biceps jerk |
| C6 | Elbow flexion | Biceps jerk |
| C7 | Elbow extension | Triceps jerk |
| C8 | Thumb IP joint extension; finger DIP flexion |  |
| T1 | Finger abduction and adduction |  |
| **Lumbar spine and lower limb** | | |
| L2 | Hip flexion |  |
| L3 | Knee extension | Knee jerk |
| L4 | Foot dorsiflexion and inversion | Knee jerk |
| L5 | Extension of big toe |  |
| S1 | Foot plantarflexion standing (or eversion of the foot) | Ankle jerk |
| S2 | Toe standing (or knee flexion) | Ankle jerk |

**^15^**Includes testing of **deep tendon reflexes (included in table above), clonus and babinski**

**^16^SLR: ‘**straight leg raise’ to evaluate mechanosensitivity of the sciatic nerve; the relaxed limb is passively raised by the assessor, producing hip flexion while maintaining knee extension to the onset of symptoms.

**^17^Special tests for particular structures,** such as joint or ligament integrity tests.

| **Region** | **Test** |
| --- | --- |
| **Hip** | Combined flexion/adduction |
| **Knee** | Lachman’s test for Anterior Cruciate Ligament |
|  | Posterior Draw and posterior sag sign |
|  | Medial Collateral Ligament test |
|  | Lateral Collateral Ligament test |
|  | Medial Meniscal test: McMurray’s test and joint line tenderness |
|  | Lateral Meniscal test: McMurray’s test and joint line tenderness |
| **Ankle** | Anterior draw |
|  | Inversion stress test |
| **Shoulder** | RC tear (passive> active ROM; passive ER maintained) |
|  | Frozen shoulder (External rotation lost: capsular pattern) |
|  | Supraspinatus tendinosis (painful arc or resisted elevation in abduction and 30° horizontal flexion) |
|  | Hawkins/Kennedy impingement test; painful arc |
| **ACJ** | Scarf test and palpation of ACJ |
| **Elbow** | Lateral elbow pain with grip or resisted middle finger extension |
| **Wrist** | Phalen’s test for carpal tunnel syndrome |
| **Functional tasks** | For stable neurological impairment: qualitative evaluation of functional tasks related to activities of daily living, such as transfers and gait. |

**^18^Provisional diagnosis** given the information currently available to assessor categorise participant’s presentation as either:

- Non-specific musculoskeletal disorder
  - Identified by region of disorder e.g. shoulder, anterior knee
  - And identified by nature of dysfunction e.g. pain, hypomobility (stiffness), instability
- Specific musculoskeletal disorder
  - Identify as named condition e.g. ACL tear, OA hip, recurrent shoulder dislocation
- Non-acute stable central nervous system disorder
  - Identify by previous medical diagnosis e.g. stroke, head injury
- Potentially serious condition
  - Identify indicators and classify as potentially systemic, infectious disease, unstable neurological condition or un-investigated (not yet medically diagnosed) neurological condition
